# Antimicrobial resistance in *E. Coli* of animal origin and discovery of a novel ICE mobile element in Northeast China

**DOI:** 10.1186/s12917-023-03828-5

**Published:** 2023-12-05

**Authors:** Dao mi Zhu, Qiang Ding, Peng hui Li, Yong liang Wang, Ya zhuo Li, Xuan yu Li, Gong mei Li, Hong xia Ma, Ling cong Kong

**Affiliations:** 1 College of Animal Science and Technology, Jilin Agricultural University, Xincheng Street#2888, Changchun, 130118 P.R. China; 2 The Engineering Research Center of Bioreactor and Drug Development, Ministry of Education, Jilin Agricultural University, Xincheng Street#2888, Changchun, 130118 P.R. China; 3 The Key Laboratory of New Veterinary Drug Research and Development of Jilin Province, Jilin Agricultural University, Xincheng #Street, Changchun, 2888, 130118 P.R. China

**Keywords:** Novel ICE, Spread of antimicrobial resistance, Antimicrobial resistance genes, Antimicrobial resistance, *E. Coli*

## Abstract

**Background:**

Multidrug resistance in *Enterobacteriaceae* including resistance to quinolones is rising worldwide. The development of resistance may lead to the emergence of new transmission mechanisms. In this study, the collection of different *E. coli* was performed from animals and subjected to subsequent procedures including pulsed-field gel electrophoresis, micro-broth dilution method, polymerase chain reaction. Whole genome sequencing of *E. coli* C3 was performed to detect the affinity, antimicrobial resistance and major carriers of the isolates.

**Results:**

A total of 66 *E. coli* were isolated and their antibiotic resistance genes, frequency of horizontal transfer and genetic environment of *E. coli* C3 were determined. The results showed there were both different and same types in PFGE typing, indicating clonal transmission of *E. coli* among different animals. The detection of antimicrobial resistance and major antibiotic resistance genes and the plasmid transfer results showed that strains from different sources had high levels of resistance to commonly used clinical antibiotics and could be spread horizontally. Whole-genome sequencing discovered a novel ICE mobile element.

**Conclusion:**

In summary, the antimicrobial resistance of *E. coli* in northeast China is a serious issue and there is a risk of antimicrobial resistance transmission. Meanwhile, a novel ICE mobile element appeared in the process of antimicrobial resistance formation.

**Supplementary Information:**

The online version contains supplementary material available at 10.1186/s12917-023-03828-5.

## Background

Antimicrobial resistance significantly threatens public health [1]. Multidrug resistance (MDR) pathogens are responsible for at least 700,000 deaths yearly [2]. *E. coli* is an important vector and potential source for the transmission of antibiotic resistance genes (ARGs) [3, 4], which are commonly transmitted to other bacteria in humans during breeding, slaughtering, and processing [5, 6]. *E. coli* has an important role in the spread of antimicrobial resistance (AMR) as a donor, recipient, and intermediate carrier of ARGs [7, 8]. In addition, bacteria can capture foreign genes through mobile elements such as mobile plasmids, integrons, mobile transposons, Integrative and Conjugative Elements (ICE), etc. [9, 10], and integrate and regulate ARGs, thereby obtaining the horizontal transfer of AMR. Therefore, the resistance carried by *E. coli* is more prone to horizontal transmission [9]. Previous studies have also reported that overuse of antibiotics, great number of animals, and low genetic diversity from intensive farming increase the risk of animal pathogens transferring to humans. For example, many kinds of *E. coli* have been detected in veterinary clinics, but their epidemic characteristics are mainly found in some intensive farms. *E. coli* not only caused certain economic losses to animal husbandry but also seriously threatened the public health safety of human beings [11].

In recent years, the antibiotic resistance of some strains, such as *methicillin-resistant Staphylococcus* [12], carbapenem-resistant *Enterobacteriaceae* [13], and MDR *Acinetobacter baumannii* [14], have attracted increasing attention. Recently, the emergence of *tet*(X4) [15] and *mcr*-1 [16] have come to pose a threat to the clinical utility of tigecycline and colistin, the last line of defense for humans.

ICE is a novel mobile element found in bacteria that can transfer between bacteria through conjugation. Many ICEs are associated with ARGs; transferring these genetic elements can accelerate the dissemination of ARGs within and between microbial genera [17]. ICE has some relatively conserved core genes, including *int*, *xis* and *tra*, whose ends are generally inverted repeats *att*L and *att*R. Also, ICE has a typical modular structure that can be divided into four modules according to the different functions mediated by gene clusters: integration and excision module (*int-xis*), mating-pair formation module (*mpf*), mobilization and processing (*mob*), and regulation (*reg*). In addition, ICE also contains some hot spots (HS) and variable regions (VR) foreign genes [18]. In 2002, complex elements that can be cut from the chromosome, integrated into the host chromosome, and transferred between bacterial cells by conjugation were first classified as ICE [19]. In the study of gram-negative intestinal bacteria, ICE R391 has raised concerns. It was found in *Providencia reitseri*, isolated from human excreta in South Africa in 1967 and initially called IncJ plasmid [20]. However, genomic analysis suggests that R391 is an ICE some 89 kb long. A large type of ICE that is more concerned by various studies is SXT [21].

The ARGs of ICE have some association with transposons, and some ICE contain complex transposon structures composed of IS*16* or IS*26*, which surround the ARGs [22]. In their study, Beaber et al. found that the widespread use of quinolone antibiotics increases the frequency of associated ICE transfer [23]. As a result, the frequency of ICE-carrying bacteria in the clinic and the environment gradually increases. Consequently, Bi and his team created ICEberg (http://db-mml.sjtu.edu.cn/ICEberg/), an integrated conjugation element database, which includes 428 ICEs in 333 bacterial strains identified through bioinformatics prediction and literature mining [24].

The World Health Organization (WHO) has classified fluoroquinolones as “critically important antimicrobials” because of their broad-spectrum effects and clinical importance in human and animal medicine [25]. Some studies have shown that minimal inhibitory concentration (MIC) **≥** 32 mg/L is a high-level resistant strain [26, 27]. In this study, an MDR *E. coli* C3 highly resistant to multiple antibiotics was isolated from *E. coli* isolated from different animals in northeast China. Whole genome analysis showed that this strain carries multiple ARGs and the novel ICE mobile element (using the database created by Bi et al.), which could be actively transmitted between bacteria through plasmids and ICE.

## Results

### Separation and identification

**Fig. 1:**
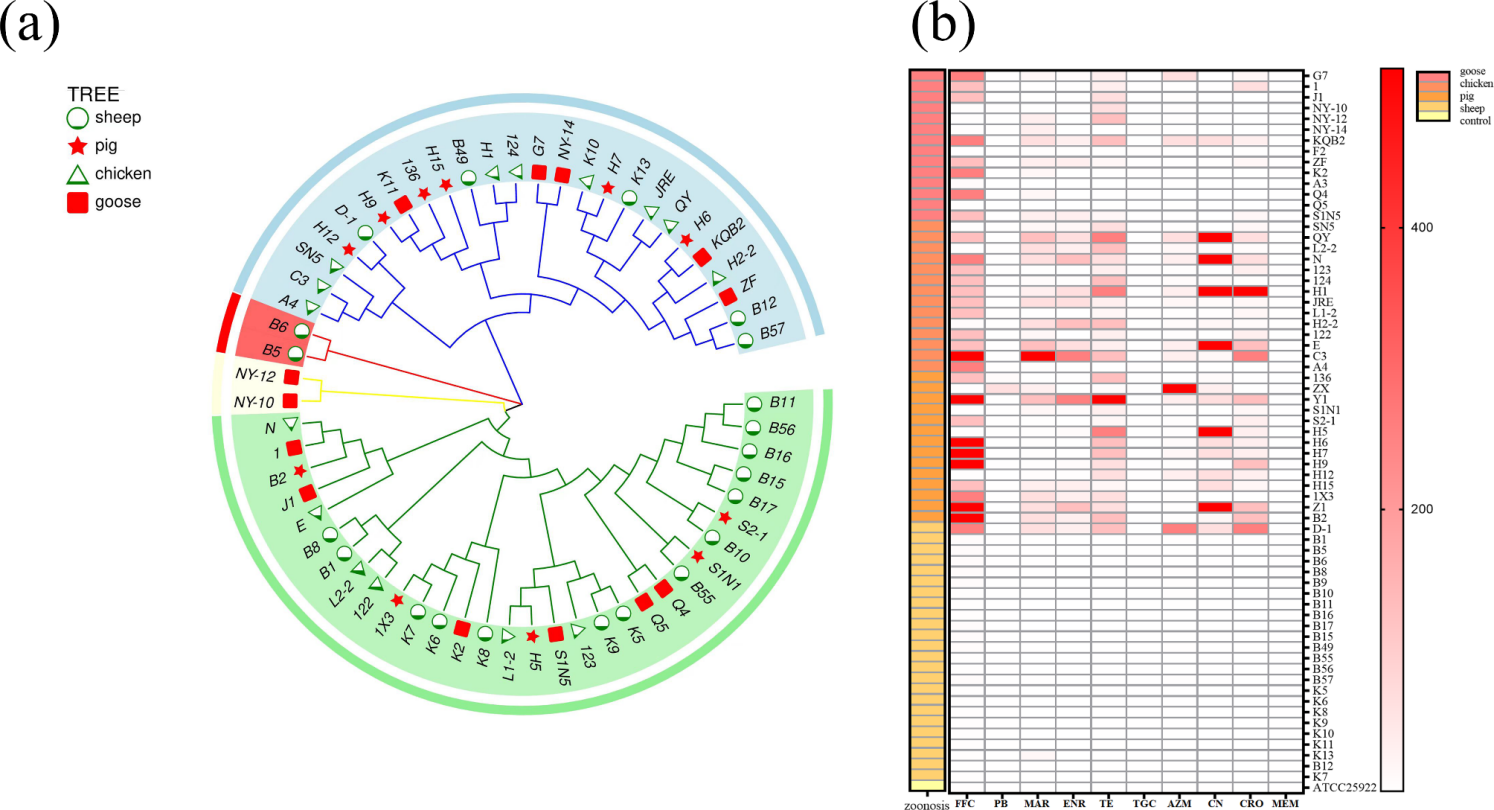
Phylogenetic tree of *E. coli* isolates and AMR detection. (**a**) Phylogenetic tree of 66 *E. coli*. (**b**) The result of antimicrobial susceptibility testing. FFC, florfenicol; PB, polymyxin B; MEM, meropenem; TGC, tigecycline; MAR, marbofloxacin; TE, tetracycline; AZM, azithromycin; ENR, enrofloxacin; CN, gentamicin; CRO, ceftriaxone

The Molecular Evolutionary Genetics Analysis (MEGA6.0) software was used to analyze the evolution based on the 16s RNA sequences of *E. coli* and standard strain *E. coli* ATCC® 25,922™ isolated from different animal sources. The results are shown in Fig. 1a. Through the construction and analysis of the evolutionary tree, 66 *E. coli* were classified into two large branches, among which different animals as a source for *E. coli* belonged to the same branch and showed high homology.

### Resistance to antimicrobial agents

**Fig. 2:**
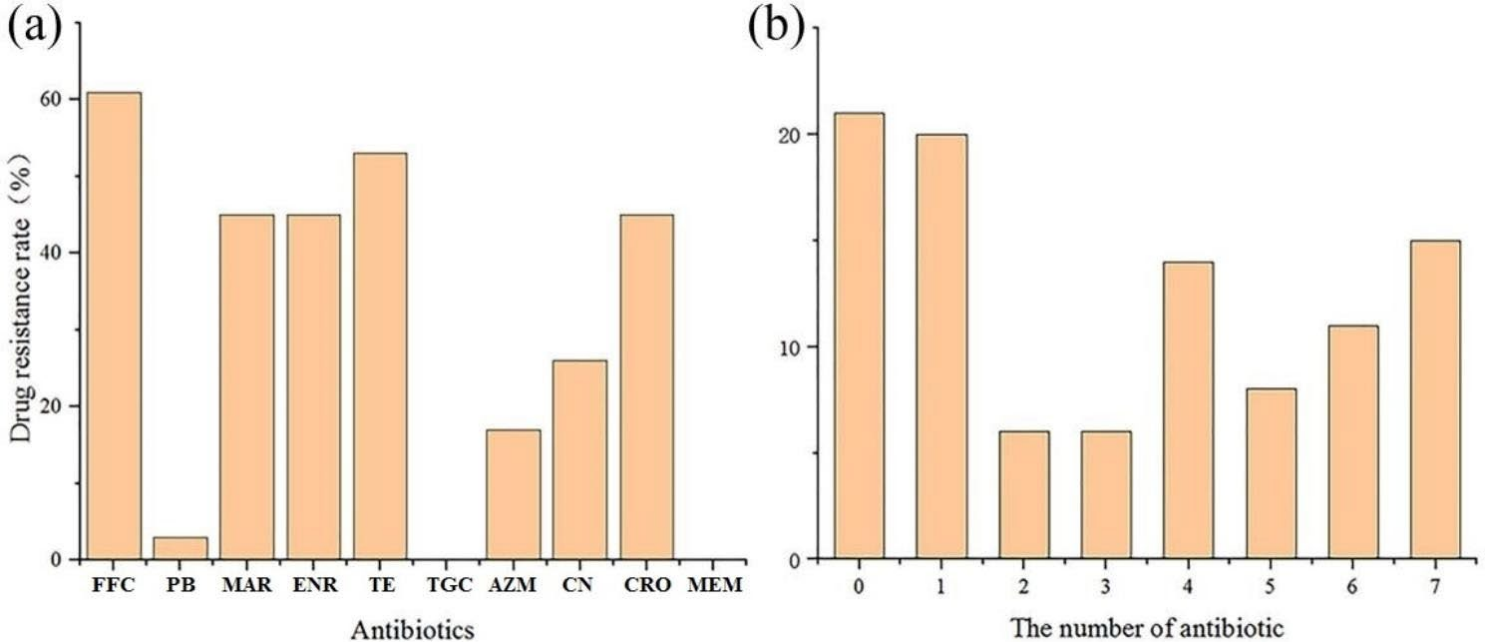
Statistical analysis of antimicrobial susceptibility testing results of the isolates. (**a**) Resistance rate of the isolates to different antimicrobial agents. (**b**) Distribution of MDR strains (%). FFC, florfenicol; PB, polymyxin B; MEM, meropenem; TGC, tigecycline; MAR, marbofloxacin; TE, tetracycline; AZM, azithromycin; ENR, enrofloxacin; CN, gentamicin; CRO, ceftriaxone

Among 66 isolates, 40 were resistant to florfenicol (FFC), 2 were resistant to polymixin B (PB), 35 were resistant to tetracycline (TE), 30 were resistant to marbofloxacin (MAR), 11 were resistant to azaerythromycin (AZN), 17 were resistant to gentamicin (CN), 30 were resistant to ceftriaxone (CRO), and 30 were resistant to enrofloxacin (ENR) (Fig. 1b). A number of MDR strains and AMR rates are shown in Fig. 2. The highest resistance rates to FFC, TE, AZM, and CRO were found in chicken (80%, 87%, 33%, and 87%, respectively); the highest resistance rates to ENR and CN were found in pigs (71% and 53%, respectively); no strains were resistant TGC and MEM; PB-resistant strains were only found in swine sources.

### Pulsed-field gel electrophoresis (PFGE) typing of E. Coli

**Fig. 3:**
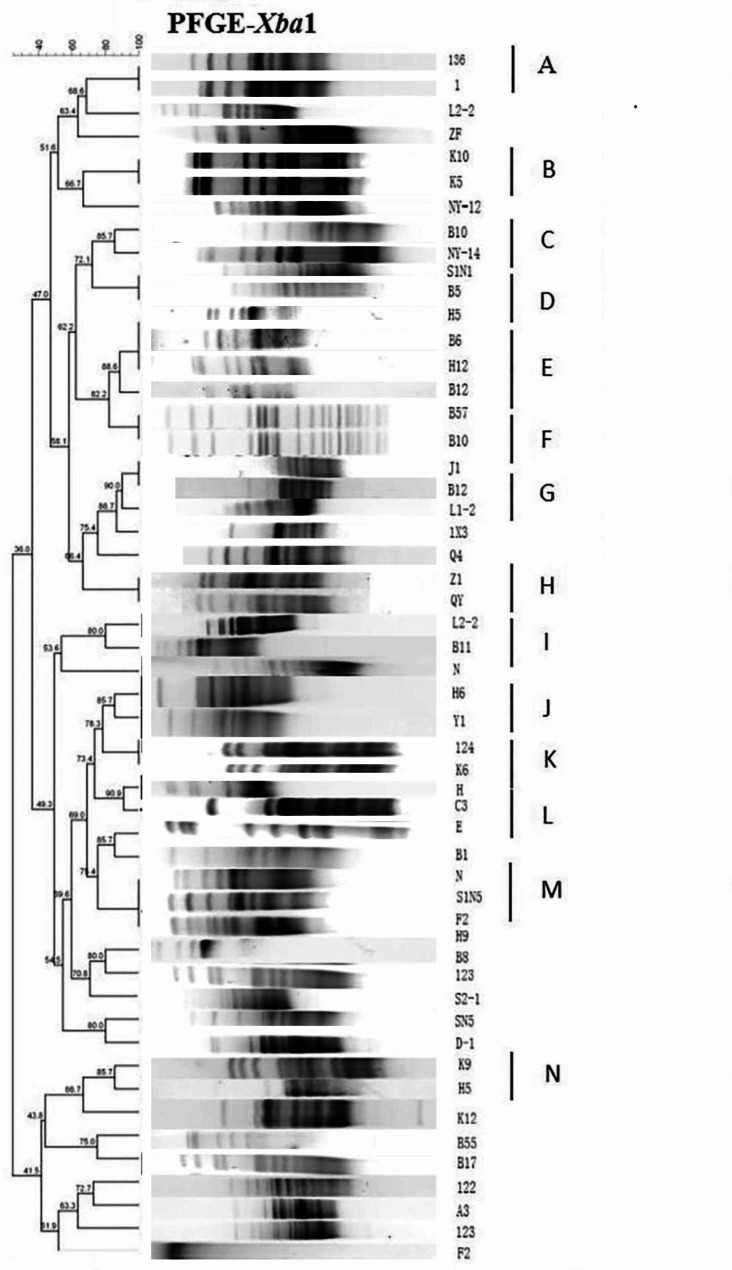
Cluster analysis of PFGE electrophoresis of *E. coli* strains. 52 *E. coli* strains were divided into 36 types. The DNA similarity of 14 clusters reached more than 85%, a total of 30 strains. The DNA map similarity of the remaining 22 strains was less than 85%, and they were divided into 22 types

After PFGE typing of 66 *E. coli*, 52 strains were successfully obtained; the *Xba1* enzyme could not digest 14 strains that were not typed. A total of 36%-100 bands were similar in 52 *E. coli* (Fig. 3). The DNA similarity of 14 clusters reached more than 85%, totaling 30 strains. In summary, 52 *E. coli* strains were divided into 36 types.

### Detection of major ARGs

**Fig. 4:**
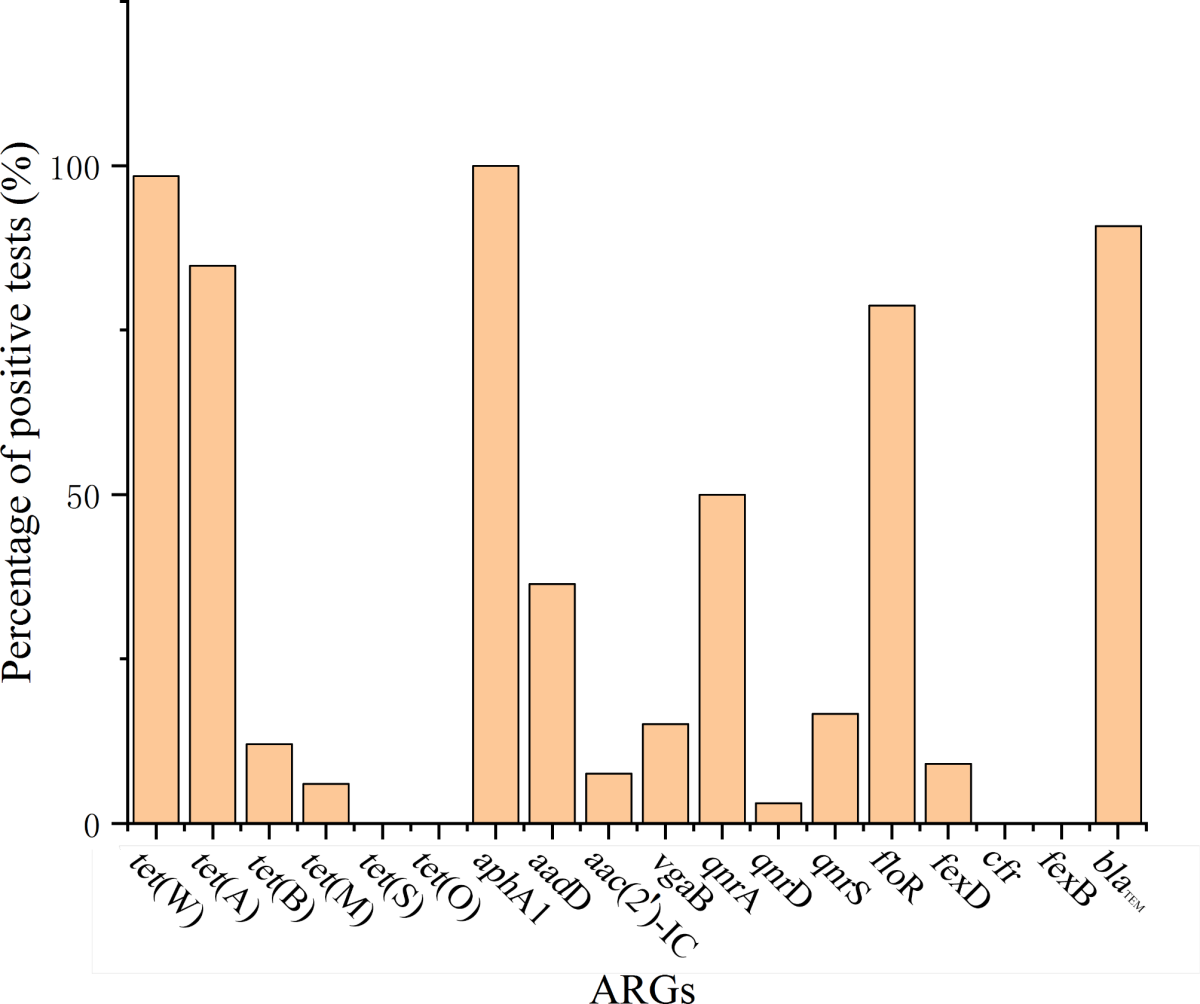
ARGs tested results of 66 isolated strains. Tetracycline resistance genes were *tet*(W) (98.5%), *tet*(A) (84.8%), *tet*(B) (12.1%) and *tet*(M) (6.0%) while *tet*(S) and *tet*(O) were not detected, aminoglycoside resistance genes were *aph*A1 (100%) and *aad*D (36.4%), *aac*(2’)-IC (7.6%), macrolide resistance gene was *vag*B (15.1%), fluoroquinolone resistance genes were *qnr*A (50.0%), *qnr*S (16.7%) and *qnr*D (3.1%), chloramphenicol resistance genes were *flo*R (78.8%) and *fex*A (9.1%) %) while *cfr* and *fex*B were not detected, β-lactam resistance genes were *bla*_TEM_ (90.9%)

The results of the major ARGs detection of 66 *E. coli* are shown in Fig. 4. The detection rates from high to low were as follows: tetracycline resistance genes were *tet*(W) (98.5%), *tet*(A) (84.8%), *tet*(B) (12.1%), and *tet*(M) (6.0%); aminoglycoside resistance genes were *aph*A1 (100%) and *aad*D (36.4%), *aac*(2’)-IC (7.6%); macrolide resistance gene was *vag*B (15.1%); fluoroquinolone resistance genes were *qnr*A (50.0%), *qnr*S (16.7%), and *qnr*D (3.1%); chloramphenicol resistance genes were *flo*R (78.8%) and *fex*A (9.1%); β-lactam resistance genes were *bla*_TEM_ (90.9%).

Table 1 shows the target site detection results of 7 high-level fluoroquinolone-resistant strains with different PFGE type. There were two types of amino acid mutations in the test strains, namely *gyr*A (Ser83Leu/Asp87Asn) and *par*C (Ser80Ile/Glu84Ala) mutations.

**Table 1:** QRDR mutations of 7 fluoroquinolone-resistant E. coli strains

Strains	MIC (mg/L) of ENR^a^	*gyr*A		*par*C	
		Ser83	Asp87	Ser80	Glu84
N	128	Leu	Asn	Ile	
QY	64	Leu	Asn	Ile	
D−1	32	Leu	Asn	Ile	
Y1	256	Leu	Asn	Ile	
H1	64	Leu	Asn	Ile	Ala
E	64	Leu	Asn	Ile	
C3	256	Leu	Asn	Ile	

^a^ ENR, enrofloxacin

### Detection of class I integron and variable regions in fluoroquinolone-resistant E. Coli

Seven *E. coli* carried class I integrase genes, and the positive rate was 100%. The positive strains carrying class I integrons were amplified by variable regions; the amplified products were 725-1925 bp in size. Three ARGs boxes were found, including the resistance genes encoding dihydrofolate reductase (*dfr*A7, *dfr*A17), aminoglycoside (*add*A22 and *add*A1), chloramphenicol (*cml*A1) and rifampicin resistance (*arr-*2). One of them carried the *add*A1, *dfr*A7, *arr*-2 and *cml*A1 resistance genes at the same time, as shown in Table 2.

**Table 2:** Summary of E. coli class I integron resistance gene boxes

Integration subtype	Amplification product size(bp)	Number of strains	Gene cassette
Class1	990	3	*aad*A22
Class1	1925	1	*add*A1*-dfr*A7*-dfr*A17*-arr-*2*-cml*A1
Class1	725	1	*dfr*A7

### Plasmid transferability

After transfer by conjugation, the antimicrobial susceptibility test determined the MIC of *Salmonella* H9812-pN, *Salmonella* H9812-pY1, and *Salmonella* H9812-pC3, as shown in Table 3.

**Table 3:** MICs of strains before and after transferred by conjugation

Strains	ENR	FFC	TE	CRO	CN
*E. coli* N	128	256	64	512	512
*E. coli* Y1	256	512	512	128	64
*E. coli* C3	256	512	128	256	16
*Salmonella* H9812-pN	0.5	128	128	512	256
*Salmonella* H9812-pY1	1	256	512	256	64
*Salmonella* H9812-pC3	1	64	128	256	16
*Salmonella* H9812	< 0.125	1	< 0.125	< 0.125	< 0.125
*E. coli* HST08-pC3	1	64	256	256	16

ENR, enrofloxacin; FFC, florfenicol; TE, tetracycline; CRO, ceftriaxone; CN, gentamicin

The conjugation transfer frequency of Y1 strain plasmid, *E. coli* C3 plasmid and N strain plasmid was 3.16 × 10^− 3^, 6.42 × 10^− 2^ and 4.12 × 10^− 4^, respectively. *E. coli* C3 plasmid had the highest plasmid transfer frequency by conjugation, indicating that the plasmid contained in the *E. coli* C3 was most prone to transfer.

The plasmids extracted by C3, N and Y1 were transferred into Stellar competent cells by electroporation. The optimal electroshock conditions optimized during the experiment were plasmid-1 µL, voltage-2400 V, resistance-200Ω, and capacitance-25 µF. The results of C3 are shown in Table 3.

### Whole genome sequencing

Whole genome sequencing (WGS) information of the *E. coli* C3 is shown in Table S3. The strain had obvious advantages in carbohydrate transport and metabolism, amino acid transport and metabolism, DNA replication, recombination and repair, and energy production and conversion (Figure S1).

Next, we used CARD-RGI (Comprehensive Antibiotic Resistance Database -Resistance Gene Identifier) to align the amino acid sequence of *E. coli* C3. The results showed 71 genes related to AMR on the C3 genome, including 42 genes involved in efflux, 14 genes with altered target sites, 9 genes that produced inactivated enzymes, 2 genes involved in biofilm formation, etc. The statistical results are shown in Table S4.

Through the whole genome sequence analysis of C3, 946 virulence genes were predicted, including enterobactin *entE*, flagellar operon *flg*L, *flg*K and many others, and the homology was generally up to 100%.

**Fig. 5:**
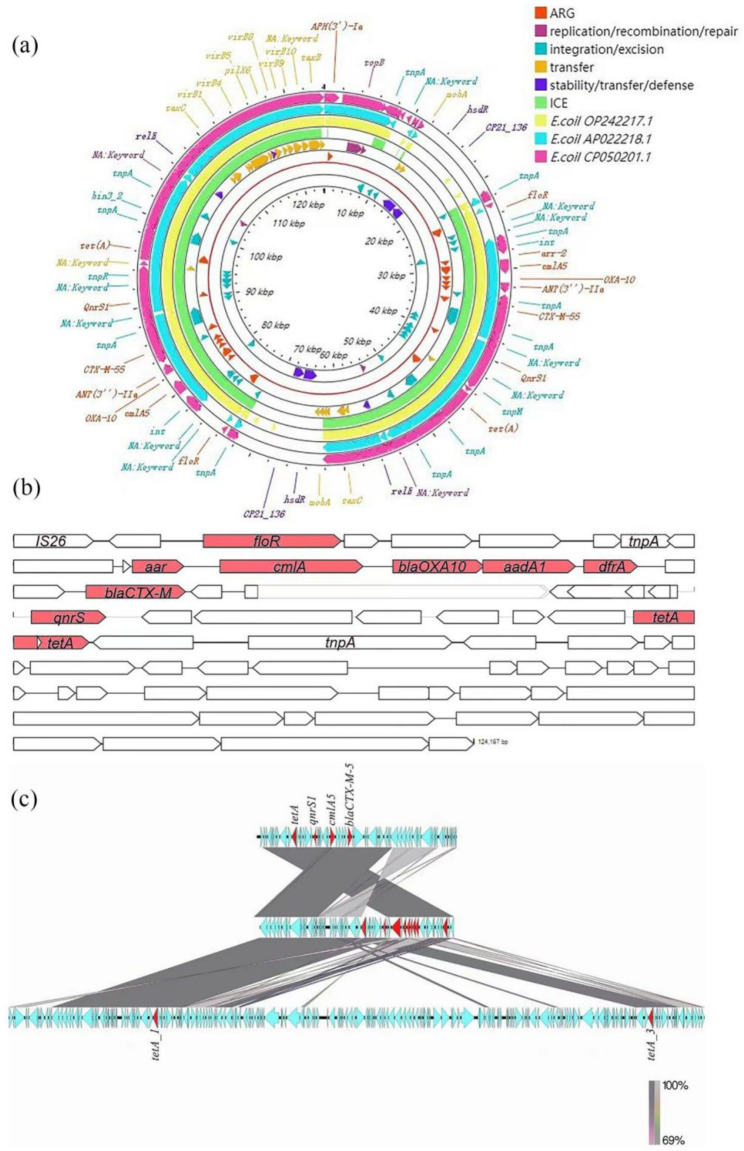
Gene distribution and ICE sequence analysis of pC3-2 plasmid in *E. coli* C3. (**a**) A 124 kb pC3-2 plasmid contained 19 ARGs, 3 gene islands, and a novel ICE mobile element. *flo*R, *cml*A, *bla*_CTX−M_, *bla*_OXA10_, *qnr*S, *tet*(A), *dfr*A and other ARGs in pC3-2 constituted an MDR gene cluster of about 27 kb. (**b**) It was found that the basic skeleton of ICE was mainly derived from the plasmid of *E. coli* AP022218.1. *tet*(A), *qnr*S, *flo*R, *cml*A, *bla*_OXA10_, *bla*_CTX−M_, *dfr*A, *aad*A1 and *arr* were located on the mobile element; The element also contained the insertion sequence IS*26* (covering all ARGs).

MGE and ICE finding online database (https://db-mml.sjtu.edu.cn/ICEfinder/) in the Center for Genomic Epidemiology database was then used to compare the whole genome sequences of *E. coli* C3. The results showed 4 ICE in *E. coli* C3, contained a novel ICE (Fig. 5). The ARGs such as *flo*R, *cmlA*, *bla*_CTX−M_, *bla*_OXA10_, *qnrS*, *tet*(A) and *dfrA* on plasmid pC3-2 formed an MDR gene cluster of about 27 kb. The related ARGs were located and found that *tet*(A) (GE004795), *qnr*S (GE004788), *flo*R (GE004769), *cml*A (GE004777), *bla*_OXA10_ (GE004778), *bla*_CTX−M_ (GE004782), *dfr*A (GE004780), *aad*A1 (GE004779) and *arr* (GE004776) were located on this mobile element. This element also contained the insert IS*26* (covering all resistance genes). Through comparison and analysis, it was found that the basic skeleton was mainly derived from the chromosome of *E. coli* AP022218.1.

## Discussion

*E. coli* of animal origin is considered an important pathogen and a causative agent for various bacterial diseases. Antimicrobial resistance (AMR) in *E. coli* is considered one of the major challenges in both animals and humans and is considered a real public health concern. Recent studies found that AMR is often acquired and disseminated through the movement of mobile genetic elements, including insertion sequences, transposons, integrons, and conjugative plasmids [28, 29]. In this study, *E. coli* was isolated from different animal sources and genotyped. The AMR analyses were performed, and resistant and virulence strains were evaluated to reflect the regional AMR status and distribution. It was found that a strain with severe resistance and a new variant gene of the ICE mobile element could mediate the horizontal spread of MDR genes.

We observed that only 21% of the isolates exhibited susceptibility to antibiotics, while 79% demonstrated resistance to at least one antibiotic, with 70% displaying MDR. Mahipal et al. found that 63.2% of the *E. coli* isolated in northern India were resistant to one or more antibiotics, of which 41% showed MDR [30]. However, Enne et al. found that only 5.7% of strains isolated from cattle (British slaughterhouse) were resistant to one or more antibiotics, while 92.1% of strains isolated from pig were resistant to at least one antibiotic [31]. Although we did not assess bovine samples, isolates from pigs were significantly larger than those from other animals, and no resistance was detected in the isolates from sheep. In addition, we did not detect TGC and carbapenem-resistant strains but found PB-resistant strains. Compared with Makarov’s study, 33% of colistin-resistant strains were higher than our results. Colistin is considered the last line of defense for human beings [32]. Moreover, our study showed that the resistance rate of FFC was the highest, followed by tetracyclines and fluoroquinolones, which was higher than that reported by Zhao (50.3% resistance to tetracyclines) and lower than that that reported by Makarov (88% restence to fluoroquinolones). Further focus should be placed on the resistance rate of tetracycline or fluoroquinolones [33].

The ARGs in the integron can be arranged in different combinations [34, 35]. It has been reported that the *dfr* (*dfr*A16, *dfr*A17) + *aad* (*aad*A1, *aad*A2, *aad*A5) gene family in the ARG boxes is the most common combination, encoding dihydrofolate reductase and aminoglyco adenosyltransferase, respectively [36]. Among them, type I integrase is the most common in MDR strains, contributing to the horizontal spread of ARGs in different genera, animals, humans and the environment [37]. Numerous studies have suggested that ARGs can be transferred through different transferable elements, such as insertion sequence (IS), transposon (Tn), integrin, plasmid, etc. [38]. Thus, in this study, conjugative transfer and electroporation experiments were performed to determine whether these resistance genes are located on the mobile plasmids. Our results showed that 3 of the 7 strains could be conjugated successfully, and *E. coli* C3 showed the highest plasmid conjugation transfer frequency of 6.42 × 10^− 2^, suggesting that the plasmids’ horizontal spread in *E. coli* C3 is fast and wide. Therefore, we electroporated the *E. coli* HST08 strain, and the MIC results were the same as those of the zygote.

An MDR *E. coli* C3 was highly antibiotic-resistant and was subject to horizontal gene transfer. Next, the WGS of the strain was performed to assess the cause of AMR transfer in *E. coli* C3. The two plasmids were typed, pC3-1 plasmid was IncFIB (similarity 98.39%) or IncFIC (similarity 95.79), and plasmid pC3-2 (Fig. 5a, Fig. S3b) was IncX1 (similarity 98.93%) [39]. Nineteen ARGs were located on the plasmid pC3-2, including *floR*, *cmlA*, *bla*_CTX−M_, *bla*_OXA10_ and *qnr*S, etc., which can mediate β-lactams, chloramphenicol, tetracyclines, quinolones and other antibiotics, and formed an ARG cluster of about 27 kb in the plasmid pC3-2. The novel ICE (Fig. 5c, Fig. S2, Fig. S3a) carried by the plasmid pC3-2 contained 9 ARGs, including *flo*R, *cml*A, *bla*_CTX−M_, *bla*_OXA10_, *qnr*S, *tet*(A), *dfr*A, *aad*A1, etc. The traceability analysis of the ICE showed that its main skeleton was derived from other *E. coli*. At the same time, the plasmid pC3-2 also carried three composite transposons IS*26*, IS*Vsa3* and IS*102*, and the transposon carried a variety of ARGs such as *bla*_CTX−M_, *bla*_OXA10_, *flo*R, *tet*(A), *aph*(3)-I, etc. The transposon IS*26*, ICE, and IS*Vsa3* all had partially overlapping ARGs. IS*102* carried ARGs similar to IS*26* and IS*Vsa3*. According to related studies, IncX-type plasmids, IS*26*, IS*Vsa3*, IS*102*, and ICE, can all mediate the horizontal transfer of ARGs [40]. For example, the ubiquitous presence of the IS element, as well as the formation and transposition of a circular IS-*tet*(X) variant intermediate, indicates the potential translocation of the *tet*(X) variant genes between different plasmids and integration into the chromosome [15].

## Conclusion

The *E. coli* from different animals in northeast China showed different levels of AMR to commonly used antibiotics, and these *E. coli* could be transmitted by cloning. At the gene level, these strains carry genes that could mediate MDR, among which *E. coli* C3 was more resistant and carried a large number of virulence genes. A novel ICE variation was found in the bacterial evolution process of *E. coli* C3. The existence of *E. coli* C3 in the animal environment poses a serious threat to the environment and even human health and safety. Under the pressure of antibiotics, bacteria could obtain plasmids of other strains to fuse with the plasmids of this strain, carrying more ARGs, and the plasmids contained multiple IS insertion elements. These findings show the importance of rational use of antibiotics.

### Methods

#### Sample collection

A total of 280 samples were collected in northeast China from September 2019 to August 2021, including 60 samples from chicken, 60 from goose, 80 from pigs, and 80 samples from sheep. Sterile cotton swabs were used for fresh fecal sampling; each sample was sealed, labeled, and then returned to the laboratory for sample processing as soon as possible. After purification, 16s rRNA universal primers for polymerase chain reaction (PCR) amplification, PCR products link pMD18-T carrier, were used on a single colony. Samples were sent for sequencing to sangon biological engineering (Shanghai) co., LTD., sequencing results were analyzed using BLAST on https://blast.ncbi.nlm.nih.gov.

#### PFGE typing of E. Coli

In order to determine the genetic relatedness of the *E. coli*. Isolates were digested with the restriction enzyme *XbaI* (3µL, 15U/µL). The gels were run at 6.0 V/cm with an initial/final switch time of 2 s/60 sec at an angle of 120° at 0° for 20 h. The *Salmonella* serovar Braenderup H9812 standard served as size markers [41]. Specific experimental methods were performed as previously described [42].

#### Antimicrobial susceptibility testing

The isolates were subjected to antimicrobial susceptibility testing by the broth microdilution method, as described by Clinical and Laboratory Standards Institute (CLSI 2019) guidelines. *E. coli* ATCC® 25,922™ was used as a quality control strain. The results were interpreted with reference to CLSI 2019. For ENR and FFC, Veterinary Microbiology Laboratory Standards were applied [43].

#### Detection of major ARGs

Polymerase chain reaction (PCR) was used to detect the respective ARGs (Table S1) of chloramphenicol, macrolides, quinolones, β-lactams, tetracyclines, and aminoglycosides, which are widely used in animals. *gyr*A and *par*C targets in the quinolone resistance determining region (QRDR) of 7 strains with different PFGE types and high fluoroquinolone resistance [27] (MIC ≥ 32 mg/L) were detected. The *gyrA* and *parC* sequences were compared with those of *E. coli* K12 available in the NCBI database.

#### Detection of class I integron and variable region in fluoroquinolone-resistant E. Coli

A total of 7 with high fluoroquinolone resistance of different PFGE types integrase positive strains were detected by PCR. Identification of the variable region of class 1 integrons was detected in the case of integrase positive. The PCR products were recovered, ligated and transformed, and the plasmids were extracted. The plasmids were sent to Shanghai BioEngineering Co., Ltd. for sequencing. Results of the sequences in the integron were compared with those in the GenBank database (NCBI).

#### Plasmid transferability

The plasmid containing integron was used for plasmid transfer by conjugation or electroshock transformation. *Salmonella* H9812 was used as a recipient. The screening was performed using an SS medium containing 0.25 mg/L enrofloxacin. The results of conjugative plasmid transfer were determined by PCR amplification detection with *Salmonella*-specific primer *JY27*, plasmid extraction, and AMR spectrum changes, and calculated according to the formula:

$$f=\frac{C\times 10X}{D\times 10Y}$$

where *f* is the conjugative transfer frequency, C is the zygote number, D is the donor number, X is the zygote dilution factor, and Y donor is the dilution factor.

Plasmids from multiple highly resistant *E. coli* were transferred into Stellar (*E. coli* HST08) competent cells by electroporation.

#### WGS

In order to analyze the gene-environment, the whole genome of *E. coli* C3 with high AMR and high transferability frequency was sequenced. Firstly, the genomic DNA was extracted, after which the purity, concentration and integrity were checked by Nanodrop, Qubit, and 0.35% agarose gel electrophoresis. Next, large fragments of DNA were recovered by the BluePippin automatic nucleic acid recovery system. After end repair, the DNA fragments were purified with magnetic beads to construct a Qubit library and sequenced using the Nanopore platform.

Reads were filtered for low-quality, adapters, and too short fragments (< 2000 bp in length) and applied to de novo assembly for error correction of assembled draft genomes. Canu v1.5 [44] software was used to assemble the filtered reads; Racon v3.4.3 software was used to correct the assembly results with third-generation reads, and Circlator v1.5.5 software to circulate and adjust the start site. Genome component analysis mainly included gene coding, non-coding RNA, gene island, CRISPR, and other analysis, while functional annotation mainly included general databases, such as nr, Uniprot, COG, KEGG and other databases, as well as CAZyme, PHI, CARD, and other proprietary databases annotation.

#### Statistical analysis

The phylogenetic tree model was constructed using MEGA6.0 based on the 16s rRNA sequences of the isolated strains. PFGE was analyzed using Bionumerics 8.0 software to characterize strain kinship. Snapgene software was used to conduct multiple alignment analyses of amino acid sequences, and the QRDR target mutations were obtained. The sequenced genes were processed using DNASTAR software.

### Electronic supplementary material

Below is the link to the electronic supplementary material.

Supplementary Material 1

Supplementary Material 2

## Data Availability

All data generated or analysed during this study are included in this published article [and its supplementary information files].

## Abbreviations

AMR: Antimicrobial resistance
MDR: Multidrug-resistant
WGS: Whole-genome sequencing
ARG: Antibiotic resistance gene
ARB: Antibiotic resistant bacteria
ICE: Integrative and conjugative element
PFGE: Pulsed-field gel electrophoresis
FFC: Florfenicol
TE: Tetracycline
CRO: Ceftriaxone
ENR: Enrofloxacin
MEM: Meropenem
PB: Polymyxin B
TGC: Tigecycline
MAR: Marbofloxacin
AZM: Azaerythromycin
CN: Gentamicin
CLSI: Clinical and Laboratory Standards Institute
PCR: Polymerase chain reaction
QRDR: Quinolone resistance determining region
MIC: Minimum inhibitory concentration
