# Supplementary figures and images for: Antimicrobial resistance in E. Coli of animal origin and discovery of a novel ICE mobile element in Northeast China
Source: BMC Vet Res. 2023 Dec 5;19:255. doi: 10.1186/s12917-023-03828-5 (PMC10696688; doi:10.1186/s12917-023-03828-5)

**PFGE (without original image processing).**


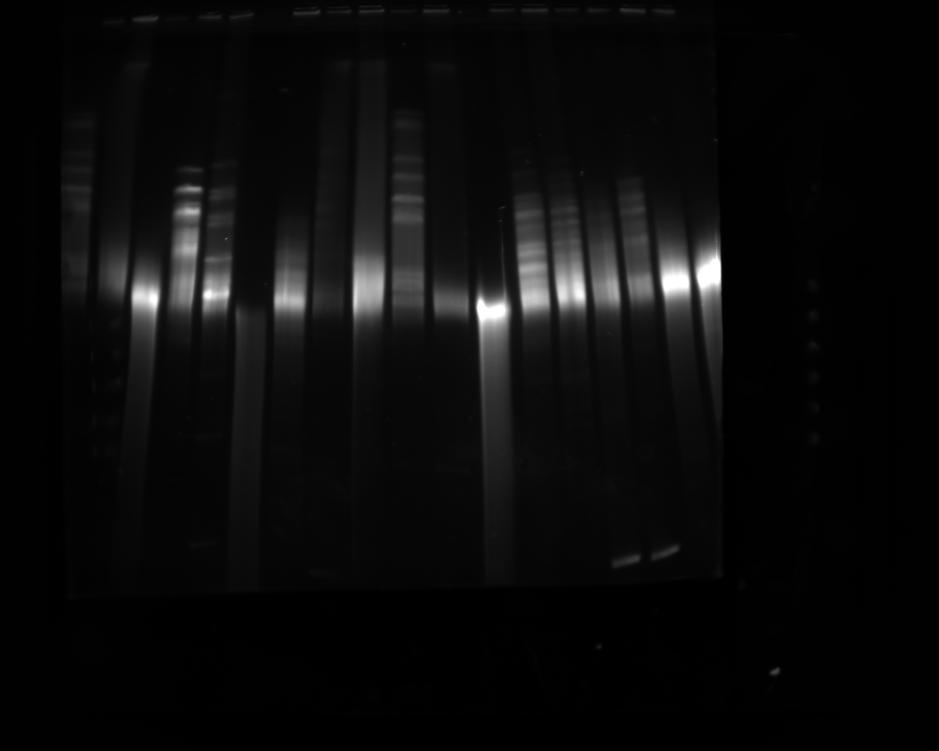


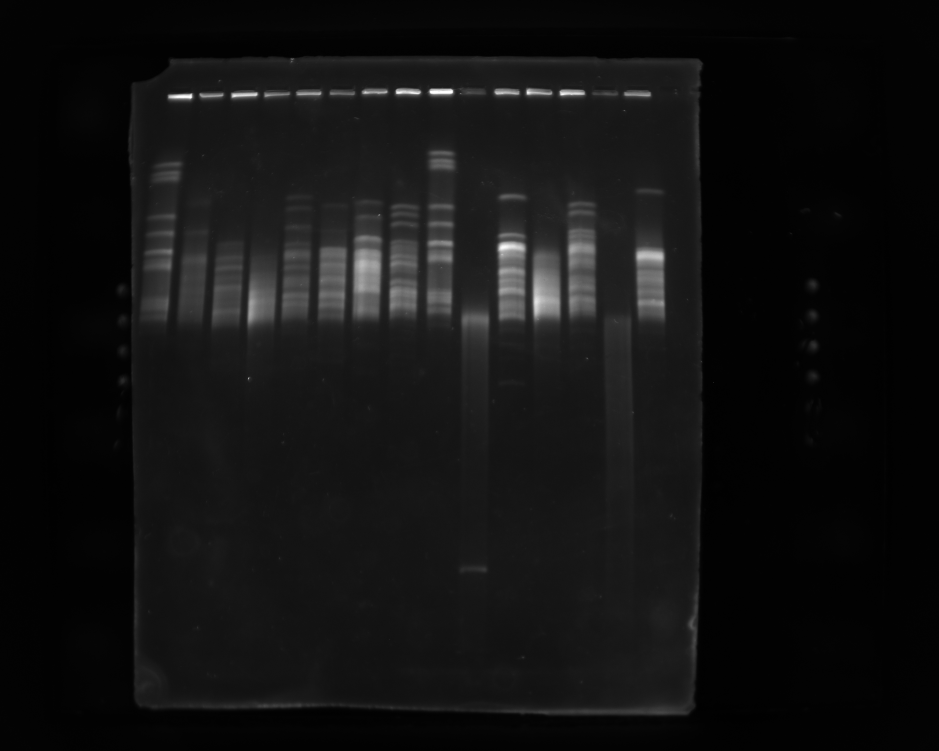


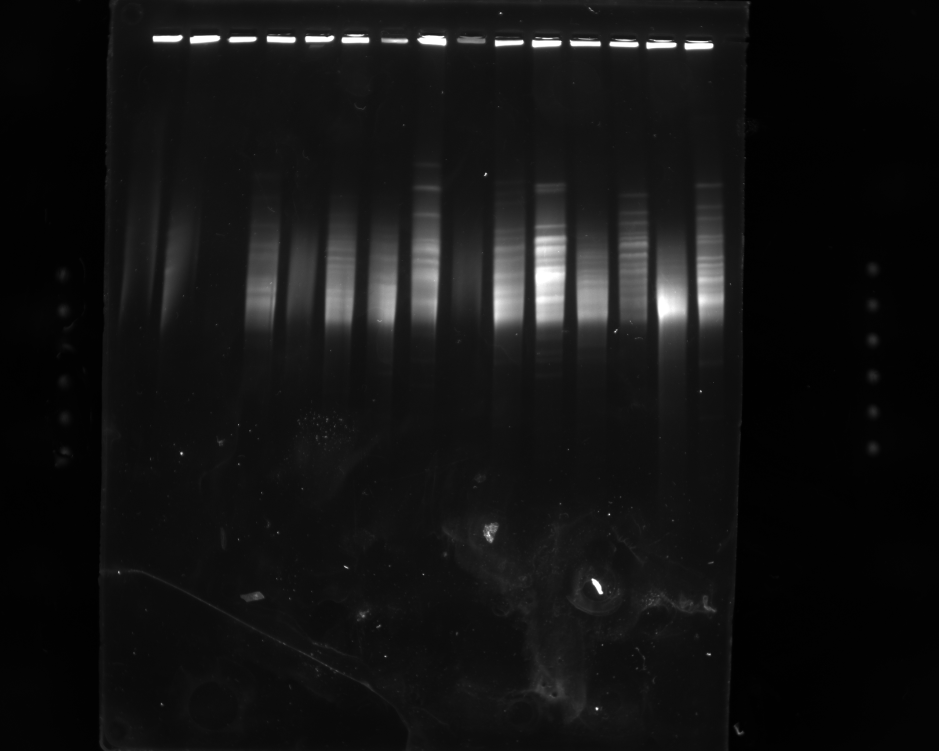


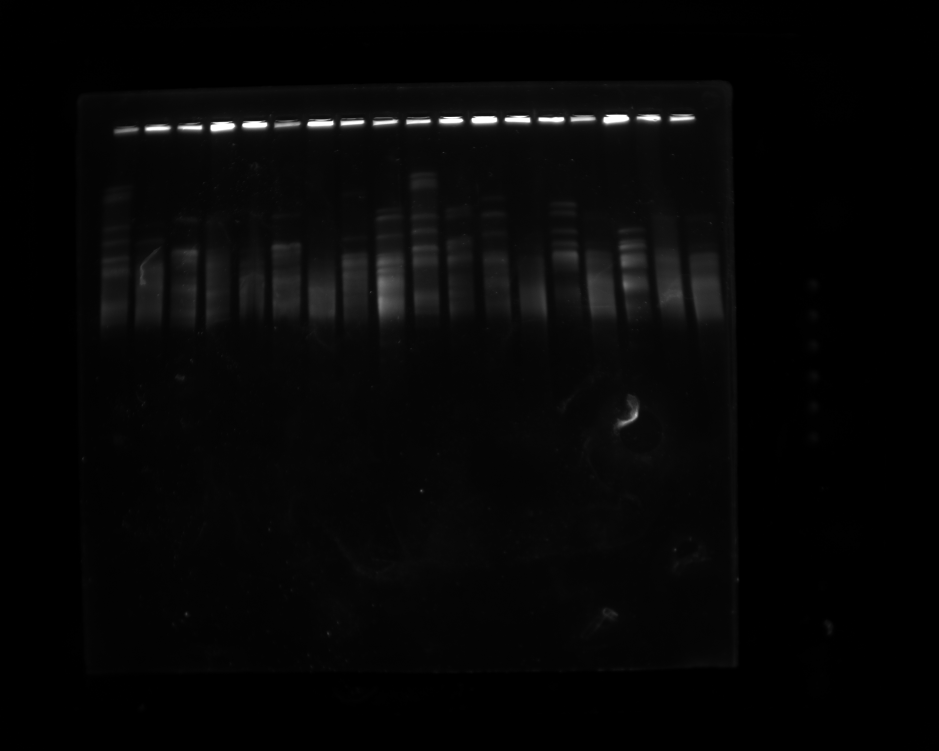


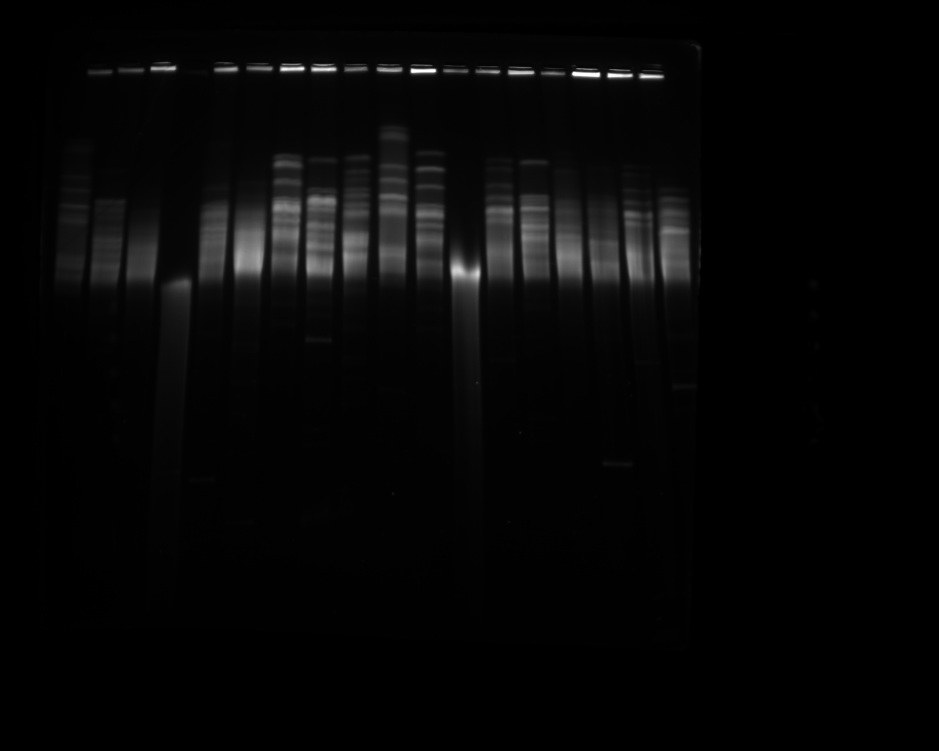

Supplement: Supplementary file 1 — Supplementary Material 1 [file 12917_2023_3828_MOESM1_ESM.docx]
