# Supplementary material for: Antimicrobial resistance in E. Coli of animal origin and discovery of a novel ICE mobile element in Northeast China
Source: BMC Vet Res. 2023 Dec 5;19:255. doi: 10.1186/s12917-023-03828-5 (PMC10696688; doi:10.1186/s12917-023-03828-5)
Supplement: Supplementary file 2 — Supplementary Material 2 [file 12917_2023_3828_MOESM2_ESM.docx]

**Antimicrobial Resistance in *E. coli* of animal origin and Discovery of a Novel ICE Mobile Element in Northeast China**

**Dao mi Zhu^1^, Qiang Ding^1^, Penghiu Li^1^, Yongliang Wang^1^, Ya zhuo Li^1^, Xuan yu Li^1^, Gong mei Li^1^, Huifeng Dong^4^, Hong xia Ma^2, 3*^, Ling cong Kong^1, 3*^**

**^1^** College of Animal Science and Technology, Jilin Agricultural University, Xincheng Street#2888, Changchun 130118, P.R. China.

**^2^** The Engineering Research Center of Bioreactor and Drug Development, Ministry of Education, Jilin Agricultural University, Xincheng Street#2888, Changchun 130118, P.R. China.

**^3^** The Key Laboratory of New Veterinary Drug Research and Development of Jilin Province, Jilin Agricultural University, Xincheng#Street 2888, Changchun 130118, P.R. China.

**^4^** Tianjin Key Laboratory of Biological Feed Additive Enterprise, S&E Burgeoning Biotechnology (Tianjin) Co., Ltd, Shengda Second Branch Road, Wangwenzhuang Industrial Park, Xiqing District, No.27, Tianjin, 300383, China

*Corresponding authors:

Hongxia Ma, E-mail: hongxia0731001@163.com

Lingcong Kong, E-mail: lingcong@jlau.edu.cn

**Table S1.** Primer amplification sequence of 20 drug resistance genes

| **Target genes** | **Primer sequences (5′- 3′)** | **Fragment length (bp)** |
| --- | --- | --- |
| *tet*(A) | F:GTAATTCTGAGCACTGTCGC  R:CTGCCTGGACAACATTGCTT | 737 |
| *tet*(B) | F:CTCAGTATTCCAAGCCTTTG  R:CTAAGCACTTGTCTCCTGTT | 416 |
| *tet*(W) | R:ATATCGGCGGAGAGCTTATCC  F:GGTGCCATTATCGGAGCAAAT | 101 |
| *tet*(M) | F:GTTAAATAGTGTTCTTGGAG  R:CTAAGATATGGCTCTAACAA | 406 |
| *tet*(E) | F:GTGATGATGGCACTGGTCAT  R:CTCTGCTGTACATCGCTCTT | 1179 |
| *tet*(O) | F:GATGGCATACAGGCACAGAC  R:CAATATCACCAGAGCAGGCT | 539 |
| *aph*A1 | F:AGCTAAGCGCGAACTGCAAT  R:TGGCTCGAAGATACCTGCAA | 67 |
| *aac*(2’)-IC | F:CAGTCGGTCTCGGTGAAGTC  F:CAGTCGGTCTCGGTGAAGTC | 101 |
| *aad*D | R:ACCGAAGCGCTCGTCGTATA  F:TACCTTATTGCCCTTGGAAGAGTTA | 101 |
| *qnr*S | F:TCGGCACCACAACTTTTCAC  R:TCACACGCACGGAACTCTAT | 255 |
| *qnr*D | F:CGAGATCAATTTACGGGGAATA  R:AACAAGCTGAAGCGCCTG | 582 |
| *qnr*A | F:GCCCGCTTCTACAATCAAGT  R:GGCAGCACTATTACTCCCAAG | 347 |
| *bla*_TEM_ | R:TCCTCCGATCGTTGTCAGAAGT  F:TTTGAAATCGGCTCAGGAAAA | 84 |
| *vag*B | F:TAAAAGAGAATAAGGCGCAAGGA  R:TGTTTAGTAGCATGTTGCATTTTCC | 97 |
| *flo*R | F:CACGTTGAGCCTCTATAT  R:ATGCAGAAGTAGAACGCG | 868 |
| *cfr* | F:TGAAGTATAAAGCAGGTTGGGAGTCA  R:ACCATATAATTGACCACAAGCAGC | 746 |
| *fex*A | F:GTACTTGTAGGTGCAATTACGGCTGA  R:CGCATCTGAGTAGGACATAGCGTC | 1272 |
| *fex*B | F:TTCCCACTATTGGTGAAAGGAT  R:GCAATTCCCTTTTATGGACGTT | 787 |
| *JY27* | F:GTGAAATTATCGCCACGTTCGGGCAA  R:TCATCGCACCGTCAAAGGAACC | 284 |
| *IntI1* | F:ACGAGCGCAAGGTTTCGGT  R:GAAAGGTCTGGTCATACATG | 249 |
| *Class I integron* | F:GCATCCTCGGTTTTCTGG  R:GGTCTGGCGGGCTTCGTG | 457 |
| *cassette* | 5’CS:GGCATCCAAGCAGCAAG  3’CS:AAGCAGACTTGACCTGA | variable |
| *gyr*A | F:ACGTACTAGGCAATGACTGG  R:AGAAGTCGCCGTCGATAGAAC | 268 |
| *par*C | F:TGTATGCGATGTCTGAACTG  R:CTCAATAGCAGCTCGGAATA | 264 |

**Table S2.** detection results of drug sensitivity of *E. coli* strains from different animals.

| **Serial number** | **FFC** | **PB** | **MAR** | **ENR** | **TE** | **TGC** | **AZM** | **CN** | **CRO** | **MEM** |
| --- | --- | --- | --- | --- | --- | --- | --- | --- | --- | --- |
| G7 | 256^a^/R^b^ | 0.5/S^b^ | 16/R | 8/R | 32/R | 0.125/S | 64/R | <0.03^a^/S | 16/R | 0.06/S |
| SN5 | 4/S | 0.5/S | 16/R | 16/R | 64/R | 0.5/S | 4/S | <0.03/S | 16/R | 0.06/S |
| 136 | 128/R | 8/R | 2/S | 1/R | 128/R | 0.125/S | 2/S | 8/I^b^ | 0.06/S | <0.03/S |
| L2-2 | 4/S | 0.5/S | 64/R | 32/R | 128/R | 0.5/S | 16/S | 8/I | 8/R | <0.03/S |
| N | 256/R | 0.5/S | 64/R | 128/R | 64/R | 0.5/S | 32/R | >512^a^/R | 64/R | <0.03/S |
| 1 | 128/R | 0.5/S | 2/S | 1/R | 32/R | 0.25/S | 1/S | 1/S | 64/R | 0.06/S |
| 123 | 128/R | 0.5/S | 0.06/S | <0.03/S | 32/R | 0.25/S | 4/S | 1/S | 32/R | 0.06/S |
| ZX | 4/S | 64/R | 32/R | <0.03/S | 8/S | 0.25/S | 512/R | 32/R | 0.5/S | 0.5/S |
| QY | 128/R | 1/S | 128/R | 64/R | 256/R | 0.25/S | 64/R | >512/R | 64/R | 0.5/S |
| D-1 | 256/R | 1/S | 64/R | 32/R | 128/R | 0.25/S | 256/R | 64/R | 256/R | 0.06/S |
| 124 | 128/R | 0.5/S | 8/R | 4/R | 128/R | 0.25/S | 8/S | 8/I | 4/R | 0.06/S |
| Y1 | 512/R | 0.5/S | 128/R | 256/R | 512/R | 0.5/S | 32/R | 64/R | 128/R | 0.06/S |
| HI | 128/R | 0.5/S | 32/R | 64/R | 256/R | 0.25/S | 32/R | >512/R | >512/R | 0.25/S |
| JRE | 128/R | 1/S | 64/R | 64/R | 32/R | 0.5/S | 16/S | 8/I | 16/R | 0.06/S |
| L1-2 | 128/R | 0.5/S | 0.06/S | <0.03/S | 16/R | 0.125/S | 2/S | 0.25/S | 16/R | 0.06/S |
| S1N1 | 2/S | 0.5/S | 16/R | 8/R | 32/R | 0.25/S | 4/S | <0.03/S | 16/R | 0.06/S |
| S2-1 | 128/R | 0.5/S | 0.06/S | <0.03/S | 16/R | 0.25/S | 4/S | <0.03/S | 32/R | 0.06/S |
| H2-2 | 8/R | 0.5/S | 64/R | 128/R | 128/R | 0.25/S | 8/S | 16/R | 8/R | 0.06/S |
| 122 | 128/R | 0.5/S | 16/R | <0.03/S | 32/R | 0.25/S | 4/S | 1/S | 32/R | 0.06/S |
| HRG5 | 4/S | 0.5/S | 4/I | 8/R | 256/R | 0.25/S | 4/S | 512/R | 32/R | <0.03/S |
| HRG6 | 512/R | 0.5/S | 4/I | 2/R | 128/R | 0.25/S | 8/S | 64/R | 32/R | 0.06/S |
| HRG7 | 512/R | 0.5/S | 4/I | 2/R | 128/R | 0.25/S | 16/S | 64/R | 32/R | 0.06/S |
| HRG9 | 512/R | 0.5/S | 4/I | 8/R | 64/R | 0.25/S | 4/S | 1/S | 128/R | 0.06/S |
| HRG12 | 0.5/S | <0.03/S | 4/I | 1/R | 64/R | 0.25/S | 32/R | 64/R | 32/R | <0.03/S |
| HRG15 | 128/R | 0.5/S | 32/R | 32/R | 16/R | 0.25/S | 4/S | 64/R | 16/R | 0.06/S |
| 1X3 | 256/R | 0.5/S | 64/R | 32/R | 64/R | 0.25/S | 4/S | <0.03/S | <0.03/S | <0.03/S |
| J1 | 128/R | 0.5/S | 1/S | 1/R | 64/R | 0.5/S | 4/S | <0.03/S | <0.03/S | 0.125/S |
| NY-10 | 4/S | 0.5/S | 2/S | 1/R | 64/R | 0.25/S | 4/S | <0.03/S | <0.03/S | <0.03/S |
| NY-12 | 4/S | 0.25/S | 32/R | <0.03/S | 128/R | 0.25/S | 4/S | 1/S | <0.03/S | <0.03/S |
| NY-14 | 4/S | 0.5/S | 32/R | <0.03/S | <0.03/S | 0.25/S | 2/S | <0.03/S | <0.03/S | <0.03/S |
| KQB2 | 256/R | 0.5/S | 64/R | 32/R | 128/R | 0.25/S | 64/R | 64/R | 32/R | 0.06/S |
| F2 | 0.5/S | <0.03/S | 4/I | 0.125/S | 0.5/S | 0.125/S | 2/S | <0.03/S | 2/I | <0.03/S |
| E | 128/R | 0.5/S | 128/R | 64/R | 32/R | 0.25/S | 32/R | >512/R | 128/R | 0.5/S |
| ZF | 128/R | 0.5/S | 32/R | 32/R | 16/R | 0.5/S | 2/S | 1/S | 16/R | 0.06/S |
| C3 | 512/R | 0.5/S | 512/R | 256/R | 128/R | 0.5/S | 32/R | 16/R | 256/R | 0.06/S |
| Z1 | 512/R | 0.5/S | 64/R | 128/R | 64/R | 0.5/S | 4/S | >512/R | 128/R | 0.06/S |
| B2 | 512/R | 1/S | 64/R | 32/R | 128/R | 0.5/S | 4/S | 16/R | 128/R | <0.03/S |
| B1 | 8/R | 0.5/S | 0.06/S | <0.03/S | 0.5/S | 0.125/S | 4/S | 1/S | 0.06/S | 0.125/S |
| B5 | 8/R | 0.5/S | 0.06/S | <0.03/S | 0.5/S | 0.125/S | 1/S | 0.5/S | <0.03/S | 0.125/S |
| B6 | 8/R | 0.5/S | 0.06/S | <0.03/S | 0.5/S | 0.125/S | 1/S | 0.5/S | <0.03/S | 0.125/S |
| B8 | 8/R | 0.5/S | 0.06/S | <0.03/S | 0.5/S | 0.125/S | 4/S | 0.5/S | <0.03/S | 0.125/S |
| B9 | 8/R | 0.5/S | 0.06/S | <0.03/S | 0.5/S | 0.125/S | 4/S | 0.5/S | <0.03/S | 0.125/S |
| B10 | 4/S | 0.25/S | 0.06/S | <0.03/S | 0.5/S | 0.125/S | 4/S | 0.5/S | <0.03/S | 0.125/S |
| B11 | 4/S | 0.5/S | <0.03/S | <0.03/S | 0.5/S | 0.125/S | 2/S | 0.5/S | <0.03/S | 0.125/S |
| B16 | 4/S | 0.5/S | <0.03/S | <0.03/S | 0.5/S | 0.125/S | 2/S | 0.5/S | <0.03/S | 0.125/S |
| B17 | 8/R | 0.5/S | <0.03/S | <0.03/S | 0.5/S | 0.06/S | 2/S | 0.5/S | <0.03/S | 0.06/S |
| B15 | 8/R | 0.5/S | 0.06/S | <0.03/S | 0.5/S | 0.125/S | 2/S | 0.5/S | <0.03/S | 0.125/S |
| B49 | 8/R | 0.5/S | 0.06/S | <0.03/S | 0.5/S | 0.125/S | 4/S | 1/S | 0.06/S | 0.125/S |
| B55 | 4/S | 0.5/S | <0.03/S | <0.03/S | 0.5/S | 0.125/S | 2/S | 0.5/S | <0.03/S | 0.125/S |
| B56 | 4/S | 0.5/S | <0.03/S | <0.03/S | 0.5/S | 0.06/S | 2/S | 0.5/S | <0.03/S | 0.06/S |
| B57 | 8/R | 0.5/S | 0.06/S | <0.03/S | 0.5/S | 0.125/S | 4/S | 0.5/S | <0.03/S | 0.125/S |
| K2 | 256/R | 0.5/S | 16/R | <0.03/S | 0.5/S | 0.125/S | 2/S | <0.03/S | <0.03/S | 0.125/S |
| K5 | 4/S | 0.25/S | <0.03/S | <0.03/S | 0.25/S | 0.06/S | 2/S | <0.03/S | <0.03/S | 0.06/S |
| K6 | 4/S | 0.25/S | <0.03/S | <0.03/S | 0.25/S | 0.06/S | 2/S | <0.03/S | <0.03/S | 0.06/S |
| K8 | 4/S | 0.25/S | <0.03/S | <0.03/S | 0.25/S | 0.06/S | 2/S | <0.03/S | <0.03/S | 0.06/S |
| K9 | 4/S | 0.25/S | <0.03/S | <0.03/S | 0.25/S | 0.06/S | 2/S | <0.03/S | <0.03/S | 0.06/S |
| K10 | 4/S | 0.25/S | <0.03/S | <0.03/S | 0.25/S | 0.03/S | 2/S | <0.03/S | <0.03/S | 0.06/S |
| K11 | 4/S | 0.25/S | <0.03/S | <0.03/S | 0.25/S | 0.03/S | 2/S | <0.03/S | <0.03/S | 0.03/S |
| K13 | 4/S | 0.25/S | 16/R | <0.03/S | 0.25/S | 0.125/S | 2/S | <0.03/S | <0.03/S | 0.125/S |
| A3 | 8/R | <0.03/S | <0.03/S | <0.03/S | <0.03/S | <0.03/S | 2/S | <0.03/S | <0.03/S | <0.03/S |
| A4 | 256/R | 0.5/S | 4/I | <0.03/S | <0.03/S | 0.5/S | <0.03/S | <0.03/S | <0.03/S | 0.25/S |
| Q4 | 256/R | 0.5/S | 16/R | <0.03/S | 0.25/S | 0.5/S | <0.03/S | <0.03/S | <0.03/S | 0.125/S |
| Q5 | 2/S | 0.03/S | <0.03/S | <0.03/S | 0.25/S | <0.03/S | 2/S | <0.03/S | <0.03/S | <0.03/S |
| B12 | 2/S | 0.25/S | 0.06/S | <0.03/S | 0.25/S | 0.125/S | 2/S | <0.03/S | <0.03/S | 0.5/S |
| K7 | 4/S | 0.5/S | 0.06/S | <0.03/S | 0.25/S | 0.06/S | 2/S | <0.03/S | <0.03/S | 0.06/S |
| S1N5 | 128/R | 0.5/S | 32/R | 32/R | 16/R | 0.5/S | 2/S | <0.03/S | 16/R | 0.06/S |
| 25922 | 4 | 0.5 | 0.06 | -^a^ | 0.5 | 0.5 | 2 | 0.25 | 0.03 | 0.125 |

FFC, florfenicol; PB, polymyxin B; MEM, meropenem; TGC, tigecycline; MAR, marbofloxacin; TE, tetracycline; AZM, azithromycin; ENR, enrofloxacin; CN, gentamicin; CRO, ceftriaxone.

^a^ unit: mg/L; "-" , natural drug resistance; >512 , greater than 512 mg/L; <0.03, less than 0.03 mg/L;

^b^ S, sensitive; R, resistant; I, intermediary.


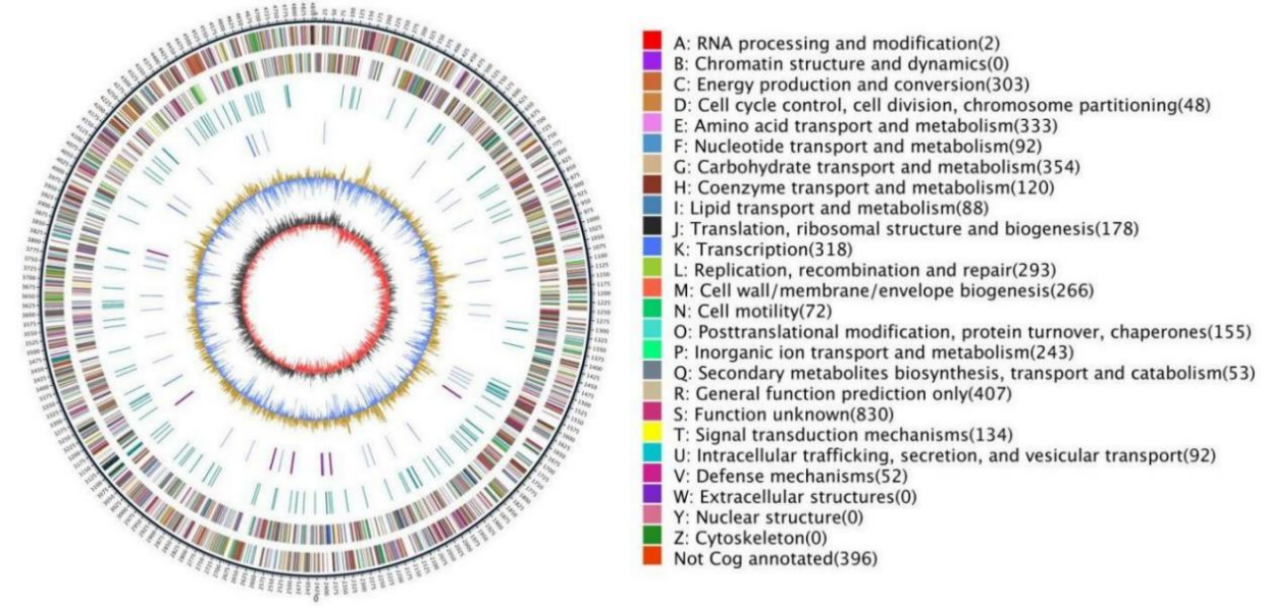


**Fig. S1.** Genome circle diagram of *E. coli* C3. The *E. coli* C3 has obvious advantages in carbohydrate transport and metabolism, amino acid transport and metabolism, DNA replication, recombination and repair and energy production and conversion, etc.

**Table S3.** Whole genome information of the *E. coli* C3

| SampleID | C3 |
| --- | --- |
| Genome size（bp） | 5,109,751 |
| Gene number | 4,829 |
| Gene total length（bp） | 4,489,998 |
| Gene average length（bp） | 929 |
| GC Content (%) | 50.86 |
| Contig Number | 3 |

**Table S4.** Major ARG primer sequences.

| GeneID | ARO_name | Resistance | Resistance Mechanism | Identities |
| --- | --- | --- | --- | --- |
| GE001025 | *kdpE* | aminoglycoside antibiotic | antibiotic efflux | 100 |
| GE001267 | *acrR* | penam; triclosan; phenicol antibiotic; cephalosporin; tetracycline antibiotic; glycylcycline; rifamycin antibiotic; fluoroquinolone antibiotic | antibiotic efflux; antibiotic target alteration | 100 |
| GE001269 | *acrB* | penam; triclosan; tetracycline antibiotic; cephalosporin; glycylcycline; fluoroquinolone antibiotic; rifamycin antibiotic; phenicol antibiotic | antibiotic efflux | 99.9 |
| GE001268 | *acrA* | penam; triclosan; tetracycline antibiotic; cephalosporin; glycylcycline; fluoroquinolone antibiotic; rifamycin antibiotic; phenicol antibiotic | antibiotic efflux | 99.75 |
| GE001689 | *pBP3* | carbapenem; cephalosporin; monobactam; penam; cephamycin | antibiotic target alteration | 99.83 |
| GE001841 | *mdtM* | acridine dye; nucleoside antibiotic; fluoroquinolone antibiotic; lincosamide antibiotic; phenicol antibiotic | antibiotic efflux | 99.76 |
| GE000194 | *marA* | penem; tetracycline antibiotic; cephalosporin; carbapenem; penam; triclosan; cephamycin; glycylcycline; monobactam; fluoroquinolone antibiotic; phenicol antibiotic; rifamycin antibiotic | antibiotic efflux; reduced permeability to antibiotic | 100 |
| GE000195 | *Escherichia coli marR* mutant conferring antibiotic resistance | penam; triclosan; phenicol antibiotic; cephalosporin; tetracycline antibiotic; glycylcycline; rifamycin antibiotic; fluoroquinolone antibiotic | antibiotic efflux; antibiotic target alteration | 100 |
| GE002075 | *eptA* | peptide antibiotic | antibiotic target alteration | 99.82 |
| GE002037 | *Escherichia coli ampC* beta-lactamase | penam; cephalosporin | antibiotic inactivation | 100 |
| GE002104 | *mdtP* | acridine dye; nucleoside antibiotic | antibiotic efflux | 100 |
| GE002103 | *mdtO* | acridine dye; nucleoside antibiotic | antibiotic efflux | 99.85 |
| GE002102 | *mdtN* | acridine dye; nucleoside antibiotic | antibiotic efflux | 99.71 |
| GE002123 | *Escherichia coli soxS* with mutation conferring antibiotic resistance | penem; tetracycline antibiotic; cephalosporin; carbapenem; penam; triclosan; cephamycin; glycylcycline; monobactam; fluoroquinolone antibiotic; phenicol antibiotic; rifamycin antibiotic | antibiotic target alteration; antibiotic efflux; reduced permeability to antibiotic | 99.07 |
| GE002122 | *Escherichia coli soxR* with mutation conferring antibiotic resistance | tetracycline antibiotic; cephalosporin; penam; triclosan; glycylcycline; fluoroquinolone antibiotic; phenicol antibiotic; rifamycin antibiotic | antibiotic target alteration; antibiotic efflux | 99.35 |
| GE002297 | *cpxA* | aminocoumarin antibiotic; aminoglycoside antibiotic | antibiotic efflux | 99.78 |
| GE002233 | *Escherichia coli EF-Tu* mutants conferring resistance to Pulvomycin | elfamycin antibiotic | antibiotic target alteration | 99.75 |
| GE002535 | *Escherichia coli UhpT* with mutation conferring resistance to fosfomycin | fosfomycin | antibiotic target alteration | 99.78 |
| GE002692 | *gadX* | penam; macrolide antibiotic; fluoroquinolone antibiotic | antibiotic efflux | 98.28 |
| GE002698 | *mdtE* | penam; macrolide antibiotic; fluoroquinolone antibiotic | antibiotic efflux | 99.74 |
| GE002697 | *mdtF* | penam; macrolide antibiotic; fluoroquinolone antibiotic | antibiotic efflux | 99.81 |
| GE002695 | *gadW* | penam; macrolide antibiotic; fluoroquinolone antibiotic | antibiotic efflux | 99.81 |
| GE002865 | *Escherichia coli EF-Tu* mutants conferring resistance to Pulvomycin | elfamycin antibiotic | antibiotic target alteration | 99.75 |
| GE002846 | *cRP* | penam; macrolide antibiotic; fluoroquinolone antibiotic | antibiotic efflux | 100 |
| GE002936 | *acrS* | penam; triclosan; tetracycline antibiotic; cephalosporin; cephamycin; glycylcycline; fluoroquinolone antibiotic; rifamycin antibiotic; phenicol antibiotic | antibiotic efflux | 99.95 |
| GE002934 | *acrF* | penam; cephalosporin; cephamycin; fluoroquinolone antibiotic | antibiotic efflux | 99.9 |
| GE002935 | *acrE* | penam; cephalosporin; cephamycin; fluoroquinolone antibiotic | antibiotic efflux | 99.74 |
| GE003156 | *tolC* | tetracycline antibiotic; aminocoumarin antibiotic; macrolide antibiotic; cephalosporin; rifamycin antibiotic; penam; triclosan; cephamycin; glycylcycline; fluoroquinolone antibiotic; phenicol antibiotic | antibiotic efflux | 99.8 |
| GE003169 | *Escherichia coli parC* conferring resistance to fluoroquinolone | fluoroquinolone antibiotic | antibiotic target alteration | 99.87 |
| GE003136 | *bacA* | peptide antibiotic | antibiotic target alteration | 99.63 |
| GE003540 | *emrR* | fluoroquinolone antibiotic | antibiotic efflux | 99.43 |
| GE003539 | *emrA* | fluoroquinolone antibiotic | antibiotic efflux | 99.74 |
| GE003538 | *emrB* | fluoroquinolone antibiotic | antibiotic efflux | 99.8 |
| GE003743 | acrD | aminoglycoside antibiotic | antibiotic efflux | 99.9 |
| GE003829 | emrY | tetracycline antibiotic | antibiotic efflux | 99.8 |
| GE003828 | *emrK* | tetracycline antibiotic | antibiotic efflux | 99.74 |
| GE003827 | *evgA* | penam; macrolide antibiotic; tetracycline antibiotic; fluoroquinolone antibiotic | antibiotic efflux | 99.51 |
| GE003826 | *evgS* | penam; macrolide antibiotic; tetracycline antibiotic; fluoroquinolone antibiotic | antibiotic efflux | 99.92 |
| GE003926 | *pmrF* | peptide antibiotic | antibiotic target alteration | 99.69 |
| GE003946 | *Escherichia coli gyrA* conferring resistance to fluoroquinolones | nybomycin; fluoroquinolone antibiotic | antibiotic target alteration | 99.89 |
| GE003959 | *yojI* | peptide antibiotic | antibiotic efflux | 99.82 |
| GE003937 | *Escherichia coli GlpT* with mutation conferring resistance to fosfomycin | fosfomycin | antibiotic target alteration | 99.78 |
| GE004127 | *baeR* | aminocoumarin antibiotic; aminoglycoside antibiotic | antibiotic efflux | 99.58 |
| GE004130 | *mdtC* | aminocoumarin antibiotic | antibiotic efflux | 99.9 |
| GE004131 | *mdtB* | aminocoumarin antibiotic | antibiotic efflux | 100 |
| GE004132 | *mdtA* | aminocoumarin antibiotic | antibiotic efflux | 99.76 |
| GE004182 | *ugd* | peptide antibiotic | antibiotic target alteration | 99.74 |
| GE004128 | *baeS* | aminocoumarin antibiotic; aminoglycoside antibiotic | antibiotic efflux | 99.79 |
| GE004564 | *sul3* | sulfone antibiotic; sulfonamide antibiotic | antibiotic target replacement | 99.62 |
| GE004559 | *mef(B)* | macrolide antibiotic | antibiotic efflux | 100 |
| GE004557 | *APH(3')-Ia* | aminoglycoside antibiotic | antibiotic inactivation | 99.63 |
| GE004659 | *TEM-1* | penam; penem; cephalosporin; monobactam | antibiotic inactivation | 99.65 |
| GE004778 | *OXA-10* | penam; cephalosporin | antibiotic inactivation | 100 |
| GE004779 | *aadA* | aminoglycoside antibiotic | antibiotic inactivation | 99.63 |
| GE004769 | *floR* | phenicol antibiotic | antibiotic efflux | 100 |
| GE004777 | *cmlA5* | phenicol antibiotic | antibiotic efflux | 99.76 |
| GE004715 | *aadA* | aminoglycoside antibiotic | antibiotic inactivation | 99.63 |
| GE004718 | *CTX-M-55* | cephalosporin | antibiotic inactivation | 100 |
| GE004724 | *qnrS1* | fluoroquinolone antibiotic | antibiotic target protection | 99.54 |
| GE004712 | *arr-2* | rifamycin antibiotic | antibiotic inactivation |  |
| GE004713 | *cmlA5* | phenicol antibiotic | antibiotic efflux | 99.76 |
| GE004714 | *OXA-10* | penam; cephalosporin | antibiotic inactivation | 99.62 |
| GE004706 | *floR* | phenicol antibiotic | antibiotic efflux | 100 |
| GE004716 | *dfrA14* | diaminopyrimidine antibiotic | antibiotic target replacement | 99.36 |
| GE004731 | *tet(A)* | tetracycline antibiotic | antibiotic efflux |  |
| GE000471 | *H-NS* | penam; macrolide antibiotic; cephalosporin; cephamycin; tetracycline antibiotic; fluoroquinolone antibiotic | antibiotic efflux | 100 |
| GE000620 | *mdtH* | fluoroquinolone antibiotic | antibiotic efflux | 100 |
| GE000632 | *mdtG* | fosfomycin | antibiotic efflux | 100 |
| GE000759 | *msbA* | nitroimidazole antibiotic | antibiotic efflux | 100 |
| GE000872 | *Escherichia coli mdfA* | benzalkonium chloride; tetracycline antibiotic; rhodamine | antibiotic efflux | 100 |
| GE000821 | *Escherichia coli nfsA* mutations conferring resistance to nitrofurantoin | nitrofuran antibiotic | antibiotic target alteration | 100 |


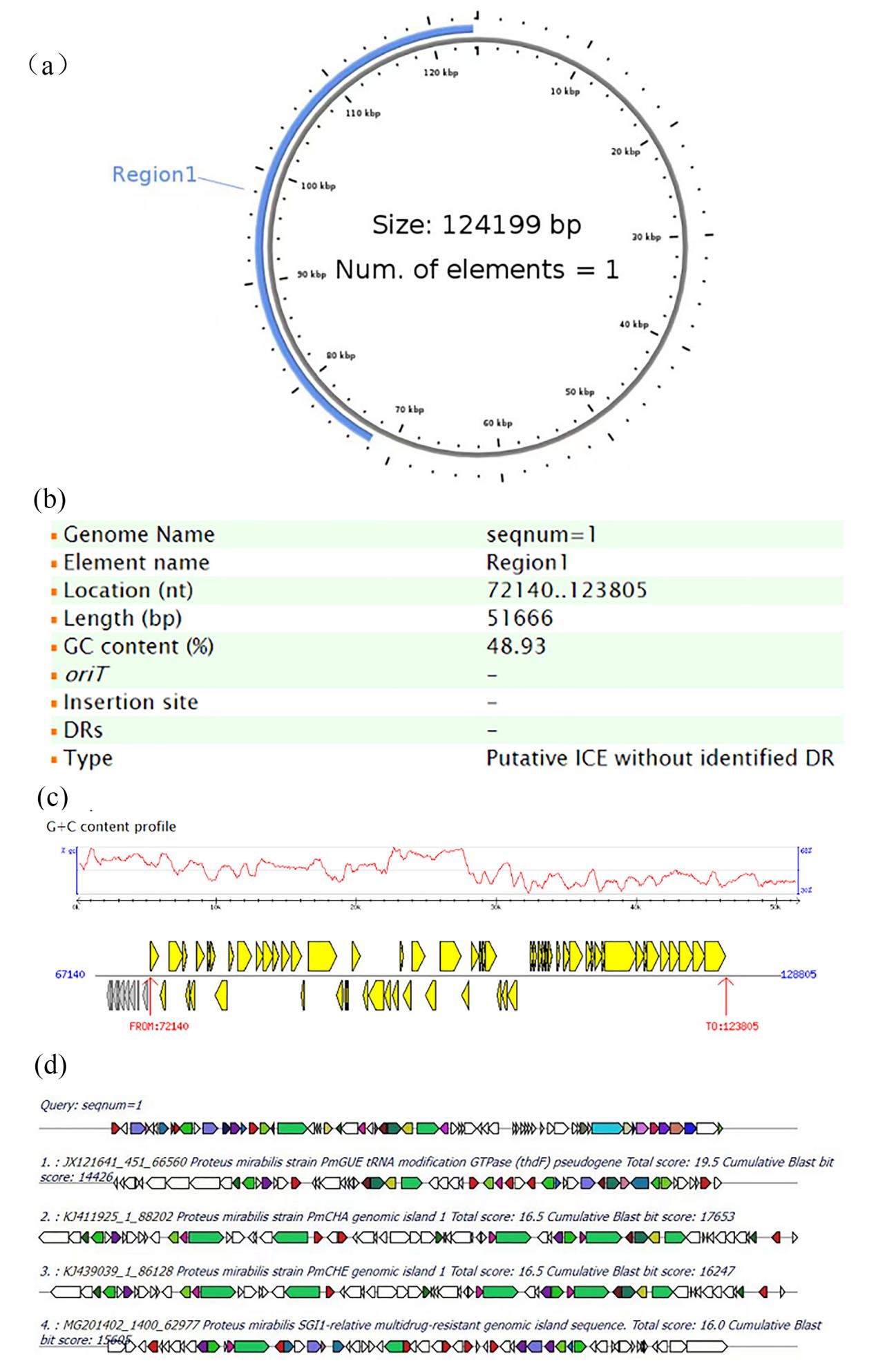


**Fig. S2.** Use ICE finder to find an untyped ICE and compare the results. (a) Utilize ICE finder to identify putative mobile elements. (b) Summary of putative mobile elements. (c) Map of putative mobile elements. (d) Results of multigene blast.


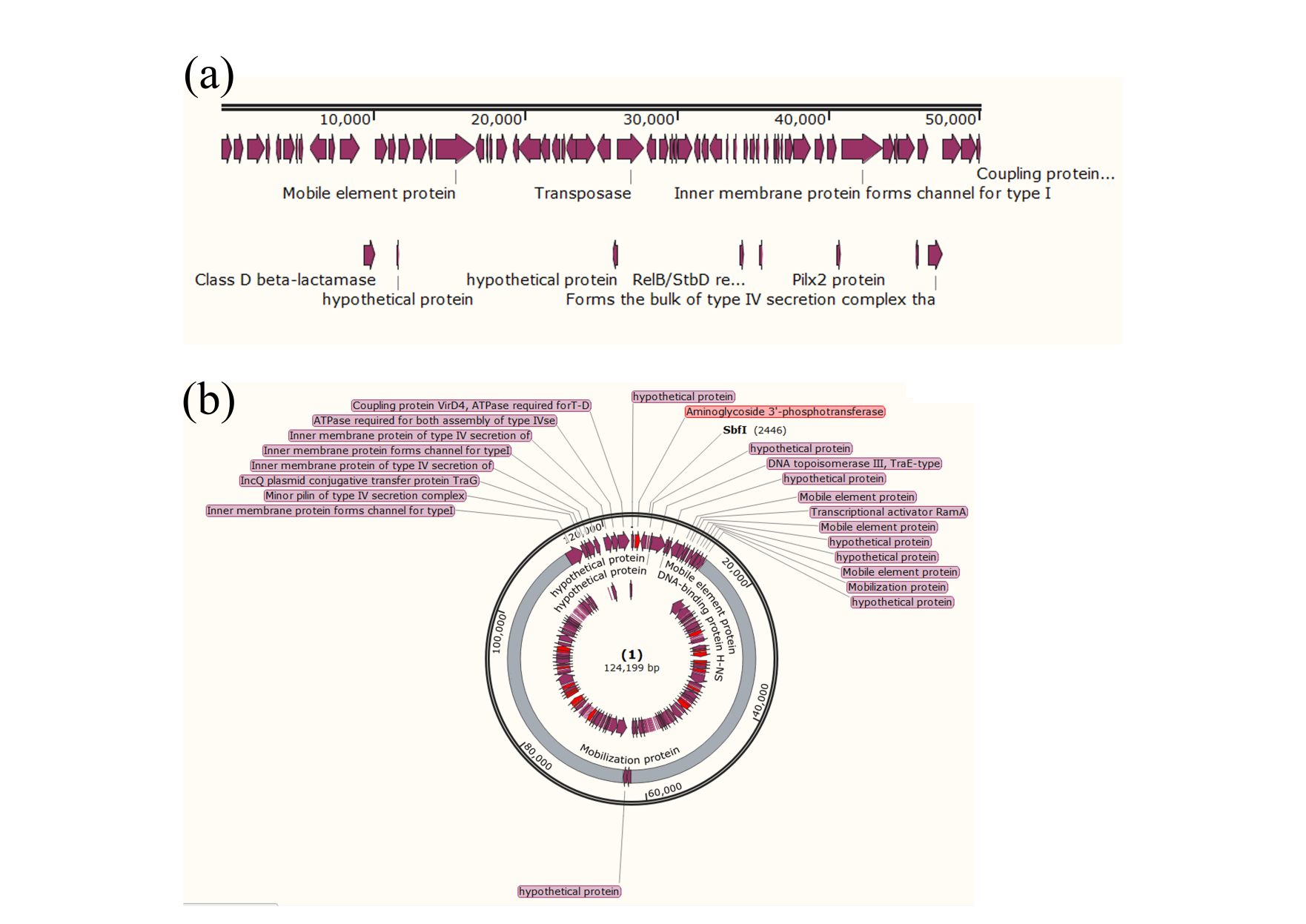


**Fig. S3.** Linear diagram of the novel ICE structure and the sequence structure diagram of plasmids in *E. coli* C3. (a) Novel linear structure diagram of ICE. (b) Structural diagram of plasmid pC3-2 of *E. coli* C3.

Materials

All antibiotic including florfenicol (FFC) (CAS: 73231-34-2), tetracycline (TE) (CAS: 60-54-8), ceftriaxone (CRO) (CAS: 73384-59-5), enrofloxacin (ENR) (CAS: 93106-60-6), meropenem (MEM) (CAS: 64221-86-9), polymyxin B (PB) (polymyxin B sulfate, CAS: 1405-20-5), tigecycline (TGC) (CAS: 220620-09-7), marbofloxacin (MBR) (CAS: 115550-35-1), azaerythromycin (AZM) (azaerythromycin A, CAS: 76801-85-9) and gentamicin (CN) (gentamycin sulfate, CAS: 1405-41-0) were purchased from Shanghai Aladdin Biochemical Technology Co., Ltd. (Shanghai, China) and Shanghai Macklin Biochemical Co. Ltd. (Shanghai, China). 1M Tris-HCL (PH8.0), 0.5M EDTA (pH8.0), SeaKem Gold agarose, X-baI enzyme, proteinase K, sodium dodecyl sulfate and disodium ethylenediamine tetraacetic acid disodium were purchased from Shanghai Bioengineering Co., Ltd. 10× Buffer, ExTaq DNA polymerase, dNTPs, XbaI and pMD18-T vector were purchased from Bao Biological Engineering (Dalian) Co., Ltd. Plasmid mini-extraction kit, bacterial DNA genome extraction kit and SanPrep column DNA gel recovery kit were purchased from Shenggong Bioengineering (Shanghai) Co., Ltd.
